# Liquid chromatography-tandem mass spectrometry technology revealed differences in serum metabolites between rheumatoid arthritis patients in different seasons

**DOI:** 10.3389/fimmu.2026.1758782

**Published:** 2026-02-10

**Authors:** Jipeng Peng, Yunyun Song, Xuemei Yuan, Feng Luo, Can Liu, Changming Chen, Qiuyi Wang, Wukai Ma, Xueming Yao

**Affiliations:** 1 Guizhou University of Traditional Chinese Medicine, Guiyang, China; 2 The Second Affiliated Hospital of Guizhou University of Traditional Chinese Medicine, Guiyang, China

**Keywords:** LC-MS/MS, metabolic differences, rheumatoid arthritis, season, serum metabolomics

## Abstract

**Objective:**

Using liquid chromatography-tandem mass spectrometry (LC-MS/MS) technology, we investigated differences in serum metabolites among rheumatoid arthritis (RA) patients in different seasons.

**Methods:**

Serum samples were collected from 60 patients meeting the diagnostic criteria for RA and divided into four groups based on different seasons. Metabolites in the serum samples were analyzed using a liquid chromatography-mass spectrometry (LC-MS) system comprising the Waters ACQUITY UPLC I-Class plus/Thermo QE plus ultra-high-performance liquid chromatography and high-resolution mass spectrometry instruments. Principal component analysis (PCA), partial least squares discriminant analysis (PLS-DA), and orthogonal partial least squares discriminant analysis (OPLS-DA) to identify seasonally differential metabolites and investigate their metabolic pathways and enrichment patterns.

**Results:**

A total of 3,787 metabolites were detected in serum, with the majority of differentially expressed metabolites classified as “Lipids and Lipid-Like Molecules.” Metabolites in the serum of RA patients across the four seasons exhibited varying degrees of differences. The significantly different metabolites identified between groups were 223 (C1_vs_C2), 977 (C1_vs _C3), and 778 (C1_vs_C4), with 62 common different metabolites among them. These differential metabolites were primarily found in the “Lipids and Lipid-Like Molecules” and “Organic acids and derivatives” categories. We also identified key differentially expressed metabolites. C1_vs_C2 includes Behenic acid (d3), Stizolamine, and Cyanidin 7-arabinoside; C1_vs_C3 includes Phosphatidylinositol-3,4,5-trisphosphate, N-Docosahexaenoyl Threonine, and Alginic acid; C1_vs_C4 includes 9,10-DiHOME, Perfluorotridecanoic acid, and 3,4-Dehydro-gamma,chi-carotene. KEGG metabolic pathway enrichment analysis showed that the differential metabolites were enriched in metabolic pathways such as Carbohydrate metabolism and Lipid metabolism.

**Conclusion:**

This study elucidates the regulatory role of seasonal factors in serum metabolic profiles of RA patients. The identified seasonally variable metabolites may provide potential reference points for seasonal monitoring of RA and optimization of personalized treatment regimens, opening new metabolomics perspectives for disease diagnosis and management.

## 1 Introduction

Rheumatoid arthritis (RA) is a common and debilitating chronic autoimmune disease with a complex pathogenesis and diverse clinical manifestations. It causes joint pain, swelling, stiffness, and can even lead to joint deformity and loss of function, causing immense physical and psychological suffering for patients. Epidemiological data show that the global prevalence of RA is approximately 0.5% to 1%, with a higher incidence in women than in men (1). For a long time, research on RA has mainly focused on fields such as immunology and pathology. However, despite the progress made by many researchers in these areas, there are still many challenges in the accurate diagnosis of the disease, monitoring of its condition, and the formulation of personalized treatment plans (2). Therefore, this study explores the serum metabolic profiles of RA patients in different seasons from a metabolomics perspective, with the aim of providing a reference for the accurate diagnosis and personalized treatment of RA.

Modern research has found that metabolic profiles vary across different seasons (3, 4), and seasonal changes may influence the physiological activities of normal organisms. Seasonal rhythms affect the immune system, with systemic inflammatory markers significantly increasing in autumn and winter. Inflammatory markers exhibit specific seasonal effects, and considering seasonal rhythms in the treatment of immune-related diseases can guide immune modulation therapy (5). In different seasons, the composition and levels of metabolites in RA patients vary.

Seasonal factors primarily regulate metabolic processes in RA patients through mechanisms such as temperature fluctuations, changes in daylight duration, and gut microbiota remodeling. Firstly, temperature variations (e.g., winter cold, summer heat) directly affect cellular membrane lipid fluidity, inducing alterations in lipase activity, thereby regulating the synthesis and degradation of core metabolites like fatty acids and glycerophospholipids (6). simultaneously disrupting intestinal barrier integrity and causing seasonal fluctuations in gut microbiota abundance and diversity. As key producers of metabolic byproducts, dysfunctions in the gut microbiota directly reduce the generation of anti-inflammatory metabolites like short-chain fatty acids and bile acids while increasing the accumulation of pro-inflammatory metabolic intermediates (7). Second, seasonal variations in daylight duration modulate the secretion rhythms of hormones like melatonin and cortisol. These hormones influence energy metabolism efficiency and the balance of inflammation-related metabolites by regulating the expression of key metabolic enzymes involved in glycolysis and lipid oxidation (8, 9). Additionally, seasonal variations in dietary composition alter the supply patterns of metabolic substrates, further affecting lipid metabolism and carbohydrate utilization in RA patients (10). The human gut microbiota maintains immune homeostasis and regulates various physiological and metabolic functions by producing metabolites such as short-chain fatty acids and bile acids. Under cold or high-temperature conditions, the gut microbiota’s responsiveness may be impaired, potentially leading to increased intestinal permeability, allowing pathogens to trigger inflammation, oxidative stress, and immune dysregulation (11). Recent studies have shown that gout exhibits seasonal trends, with changes in temperature and humidity potentially influencing uric acid (UA) levels (12). Additionally, regarding Bacillus Calmette-Guérin (BCG) vaccination, compared to spring BCG vaccination, winter BCG vaccination induces peripheral blood mononuclear cells (PBMCs) to produce more pro-inflammatory cytokines under different bacterial and fungal stimuli; conversely, winter BCG vaccination results in lower IFNγ levels released by PBMCs compared to spring BCG vaccination (13). The concentrations of interleukin-6 (IL-6) and IL-1β fluctuate seasonally. Elevated levels of these cytokines in serum may exacerbate inflammatory responses, leading to more severe RA symptoms. Simultaneously, these inflammatory mediators serve as key regulators within metabolic networks. By influencing lipid profiles, they further amplify metabolic abnormalities in RA patients (14), confirming the link between seasonal variations and human physiological activity (15). Therefore, further investigating metabolic differences in RA across different seasons can provide a basis for personalized treatment strategies for RA.

Metabolomics, as an emerging discipline, offers a novel perspective and methodology for further understanding the pathogenesis and pathophysiological processes of RA. Metabolomics enables comprehensive and systematic analysis of all small-molecule metabolites in the body, reflecting metabolic changes under specific physiological or pathological conditions. Through metabolomics technology, we can detect the levels and patterns of various metabolites in RA patients. These metabolic changes may be closely associated with multiple biological processes, including inflammatory responses, immune regulation, energy metabolism, and cellular signaling. Applying metabolomics to RA research holds promise for identifying new biomarkers, providing more sensitive and specific indicators for early diagnosis.

Although existing studies have confirmed that climate change can affect immune system function, inflammatory marker levels, and gut microbiota homeostasis (16, 17), and metabolomics has provided a novel perspective for elucidating the pathogenesis of RA (18). However, the specific mechanisms by which climate change influences RA inflammatory activity through regulation of serum metabolic networks remain unclear. Particularly lacking are systematic studies on the seasonal metabolic characteristics of RA populations in specific geographic regions. Consequently, the application of seasonal factors in personalized RA treatment lacks clear theoretical support and practical protocols, and this knowledge gap constrains the advancement of precision medicine for RA. Therefore, this study focused on RA patients in Guiyang, China. Using highly sensitive, high-throughput LC-MS/MS technology, we systematically detected and analyzed small-molecule metabolites in the serum of RA patients across spring, summer, autumn, and winter. Through multivariate statistical analysis, we screened seasonally differential metabolites, identified their category characteristics and expression patterns, and combined KEGG pathway enrichment analysis to reveal the seasonal regulatory roles of core metabolic pathways. This study may identify potential biomarkers associated with seasonal fluctuations in RA by elucidating the mechanisms through which seasonal factors influence RA serum metabolomics. It could provide partial reference indicators for seasonal disease monitoring and further enable the development of seasonally adapted, individualized treatment plans in clinical practice.

## 2 Materials and methods

### 2.1 Introduction to LC-MS/MS technology

LC-MS/MS technology is suitable for analyzing non-volatile or thermally unstable metabolites, featuring high throughput, high resolution, and high sensitivity. Ultra-high-pressure liquid chromatography or ultra-high-performance liquid chromatography uses smaller particle size chromatographic packing materials combined with higher pressures to achieve better separation, including higher separation capacity, faster separation speed, and better sensitivity. In MS scanning mode, tandem mass spectrometers can rapidly switch between high and low collision energies, simultaneously collecting first- and second-order mass spectrometry information of metabolites. Combined with metabolomics data processing software for mass spectrometry data analysis, hundreds to thousands of metabolites can be detected and identified in a single analysis. The workflow includes sample pretreatment, metabolite extraction, LC-MS/MS full-scan detection, data preprocessing, and statistical analysis.

### 2.2 Research design

**Figure 1:**
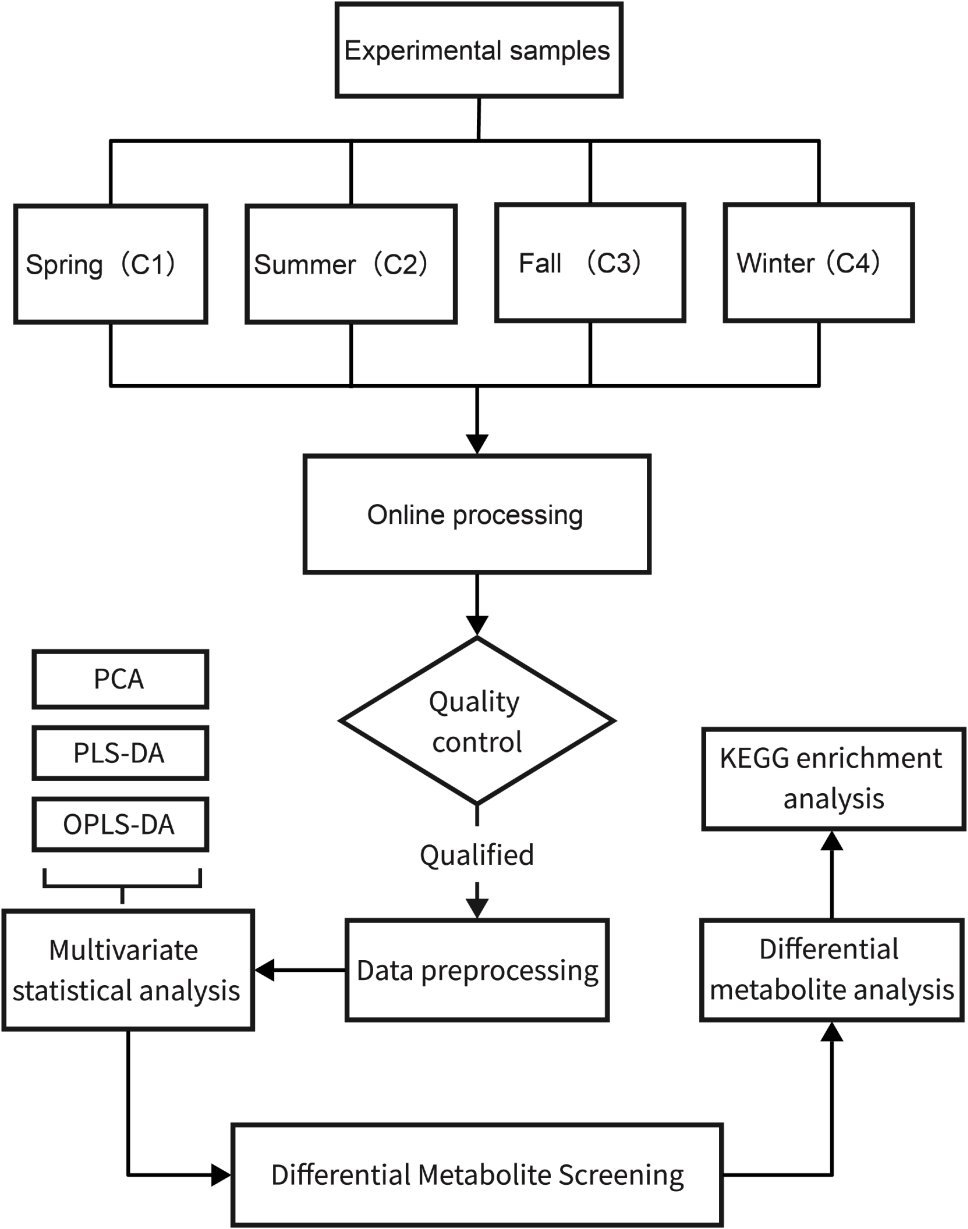
Research flowchart.

This study is a single-center, cross-sectional study. Case collection and serum sample collection were completed between September 1, 2023, and August 31, 2024. All enrolled patients voluntarily participated in the study after signing informed consent forms. The Ethics Committee of the Second Affiliated Hospital of Guizhou University of Traditional Chinese Medicine reviewed and approved the study (YJS2022220573). The study process is shown in **Figure 1**.

### 2.3 Case selection

Inclusion criteria: Meets the 2010 ACR/EULAR criteria for RA diagnosis (19); aged 18–70 years, not currently taking steroids or immunosuppressants, or has discontinued such medications for more than three months; no gender restrictions; dietary habits consistent with those of the Guiyang region; no involvement of other systems, such as pulmonary interstitial lesions; the patient voluntarily agrees to participate in this trial and signs an informed consent form. Exclusion Criteria: Patients with significant organ damage unrelated to RA or life-threatening conditions; pregnant or breastfeeding women and patients with mental disorders; patients whose dietary habits do not align with the dietary habits of the Guiyang region; patients with recent history of poor dietary habits or alcohol abuse; History of severe gastrointestinal disorders such as gastroenteritis, peptic ulcers, or gastric tumors; use of antibiotics, corticosteroids, or probiotics within the past month or two weeks; volunteers with other autoimmune diseases or patients who received anti-inflammatory treatment within the past month prior to sampling; inability or unwillingness to provide informed consent or comply with trial requirements. Grouping: All enrolled patients must have resided in Guiyang City for an extended period. Patients will be divided into four groups based on the seasons (15 patients per group, totaling 60 patients), specifically: spring (C1), summer (C2), autumn (C3), and winter (C4).

### 2.4 Sample collection

Collect 3 mL of blood using a standard serum collection tube. Allow the sample to stand at room temperature for 30 minutes, then centrifuge at 3,000 rpm for 10 minutes at 4 °C. Transfer the supernatant to labeled EP tubes, dispensing 200 μL per tube, with five aliquots retained for each sample. Immediately store the aliquots at -80 °C for future use.

### 2.5 Sample preparation

Remove the samples stored at -80 °C, thaw them in an ice-water mixture, and transfer 100 μL of the sample to a 1.5 mL EP tube. Add 400 μL of protein precipitation agent (methanol-acetonitrile, V:V = 2:1, containing mixed internal standards, 4 μg/mL), and vortex for 1 minute. Ultrasonicate in an ice-water bath for 10 minutes, then incubate at -40 °C for 2 hours. After incubation, centrifuge at high speed (13,000 rpm) at 4 °C for 20 minutes. Use a syringe to aspirate 150 μL of the supernatant, transfer it to an LC injection vial, and store at -80 °C for subsequent LC-MS analysis. Quality control (QC) samples were prepared by mixing equal volumes of extraction solutions from all samples. All extraction reagents used in sample preparation were pre-cooled at -20 °C prior to use. Detailed information on the reagents used in this experiment is provided in **Table 1**.

**Table 1:** Reagent information.

Reagent name	Catalog number	Manufacturer’s english name/Alternative name	Purity
Methanol	A452-4	fisher	HPLC, 99.9%
Acetonitrile	A998-4	fisher	HPLC, 99.95%
Formic acid	A117-50	fisher	HPLC, 99.0%
L-2-Chlorophenylalanine (Mixed Internal Standard)	C2001	Shanghai Hengchuang Bio	HPLC, 98.0%
Succinic acid-d4 (mixed internal standard)	293075-1G	Sigma	HPLC, 98.0%
L-valine-d8 (mixed internal standard)	HY-I1124	Shanghai Haoyuan Bio	HPLC, 98.0%
Cholic acid-D4 (mixed internal standard)	S22155-50mg	Shanghai Yuanye Bio	HPLC, 98.0%
D-sphingomyelin free acid (mixed internal standard)	S19260-100mg	Shanghai Yuanye Bio	BR grade, 99.0%

### 2.6 Experimental equipment and parameters

The analytical instrumentation used in this experiment was a liquid-chromatography/mass spectrometry (LC-MS) system comprising a Waters ACQUITY UPLC I-Class plus/Thermo QE plus ultra-high-performance liquid chromatography tandem high-resolution mass spectrometer, equipped with a heated electrospray ionization (ESI) source (Thermo Fisher Scientific, Waltham, MA, USA), for analyzing metabolomics in ESI positive ion and ESI negative ion modes. Instrument information is shown in **Table 2**.

**Table 2:** Instrument information.

Instrument	Model and specifications	Manufacturer
Ultrasonic Cleaner	F-060SD	Shenzhen Fuyang Technology Group Co., Ltd.
Vortex Oscillator	TYXH-I	Shanghai Hanno Instrument Co., Ltd.
Benchtop High-Speed Freezing Centrifuge	TGL-16MS	Shanghai Lu Xiangyi Centrifuge Instrument Co., Ltd.
Liquid Chromatography-Mass Spectrometry System	Waters ACQUITY UPLC I-Class plus/Thermo QE plus	Waters/Thermo
Liquid Chromatography Column	ACQUITY UPLC HSS T3 (100 mm×2.1 mm, 1.8 um)	Waters

The ACQUITY UPLC HSS T3 column (100 mm × 2.1 mm, 1.8 μm) was used in both positive and negative modes. The gradient elution system consists of (A) water (containing 0.1% formic acid, v/v) and (B) acetonitrile (containing 0.1% formic acid, v/v), with the following gradient: 0.01 min, 5% B; 2 min, 5% B; 4 min, 30% B; 8 min, 50% B; 10 min, 80% B; 14 min, 100% B; 15 min, 100% B; 15.1 min, 5% and 16 min, 5% Flow rate: 0.35 mL/min; column temperature: 45 °C. All samples were stored at 10 °C during analysis, with an injection volume of 3 μL. Details are shown in **Table 3**. The mass range was from 100 m/z to 1200 m/z. The primary mass spectrometry scan resolution was 70,000, and the secondary mass spectrometry scan resolution was 17,500, with collision energies of 10, 20, and 40 eV. The mass spectrometer operating conditions are as follows: spray voltage, 3800 V (+) and 3200 V (−); sheath gas flow rate, 35 arbitrary units; auxiliary gas flow rate, 8 arbitrary units; capillary temperature: 320 °C; auxiliary gas heater temperature, 350 °C; lens RF level, 50. Specific mass spectrometry conditions are detailed in **Table 4**.

**Table 3:** Elution gradient.

Time	A%	B%
0	95	5
2	95	5
4	70	30
8	50	50
10	20	80
14	0	100
15	0	100
15.1	95	5
16	95	5

**Table 4:** Mass spectrometry parameters.

Parameters	Positive ions	Negative ions
Spray Voltage (V)	3800	-3200
Capillary Temperature (°C)	320	320
Aux gas heater temperature (°C)	350	350
Sheath Gas Flow Rate (Arb)	35	35
Aux gas flow rate (Arb)	8	8
S-lens RF level	50	50
Mass range (m/z)	70-1050	70-1050
Full ms resolution	70000	70000
MS/MS resolution	17500	17500
NCE/stepped NCE	10, 20, 40	10, 20, 40

### 2.7 Data preprocessing and statistical analysis

LC-MS raw data were processed using Progenesis QI V3.0 software (Nonlinear Dynamics, Newcastle, UK) for baseline filtering, peak identification, integration, retention time correction, peak alignment, and normalization. Key parameters included a precursor tolerance of 5 ppm, a product tolerance of 10 ppm, and a product ion threshold of 5%. Compounds were identified using The Human Metabolome Database (HMDB), Lipidmaps (V2.3), Metlin, and an in-house database, based on precise mass-to-charge ratio (M/z), secondary fragments, and isotope distribution. The extracted data were further processed to remove any peaks with more than 50% missing values (ion intensity = 0) in the group, replace zero values with half of the minimum value, and screen based on the qualitative results of the compounds. Compounds with database match scores below 36 points (out of a total of 80 points) were also considered inaccurate and deleted. Positive and negative ion data were combined into a single data matrix.

Import the data matrix into the R package for PCA to observe the overall distribution of samples and the stability of the entire analysis process. Use OPLS-DA and PLS-DA to distinguish metabolic differences between groups. The variable importance in projection (VIP) values obtained from the OPLS-DA model were used to rank the overall contribution of each variable to group discrimination. To prevent overfitting, 7-fold cross-validation and 200 response permutation tests (RPT) were used to assess model quality. A two-tailed Student’s t-test was further used to validate whether the differences in metabolites between groups were significant. Differential metabolites with VIP values greater than 1.0 and *p*-values less than 0.05 were selected. The selected differential metabolites were annotated using the KEGG database, followed by metabolic pathway annotation to identify the pathways in which the differential metabolites were involved.

## 3 Results

### 3.1 Basic climatic characteristics

Regarding average humidity, no statistically significant differences were observed among the four seasons (P > 0.05). For average temperature, no statistically significant difference was found between the spring and autumn groups (P > 0.05), while all other intergroup comparisons yielded statistically significant differences (P < 0.05) (**Table 5**).

**Table 5:** Basic climatic characteristics.

Group	Average relative humidity (%)	Average temperature (°C)
C1	77.00(68.00, 85.75)	16.10 (12.75, 21.23)^xz^
C2	79.00(72.00, 86.00)	23.50(21.65, 24.80)^yz^
C3	74.00(67.00, 87.00)	18.00(13.80, 22.10)^z^
C4	80.00(65.00, 93.00)	6.10(3.00, 10.10)
H	3.934	222.765
P	0.269	<0.001

x compared with the summer group, P<0.05; y compared with the autumn group, P<0.05; z compared with the winter group, P<0.05.

### 3.2 Clinical characteristics of the patients

This study included a total of 60 cases, comprising 10 males and 50 females, with a male-to-female ratio of 1:7.6, indicating a higher proportion of females than males. The Spring group consisted of 15 cases (2 males, 13 females); the Summer group comprised 15 cases (2 males, 13 females); the Autumn group included 15 cases (3 males, 12 females); and the Winter group contained 15 cases (3 males, 12 females). Chi-square analysis revealed no statistically significant differences in gender distribution among the four groups (χ² = 0.48, P = 0.923). Univariate analysis of variance showed no statistically significant differences in age across the four groups (P > 0.05). There were no statistically significant differences in disease duration or body mass index (BMI) across the four seasons (P > 0.05).

Regarding DAS28-CRP scores, the spring group (5.47 ± 0.51) was higher than the summer group (4.82 ± 0.79), the spring group (5.47 ± 0.51) was higher than the autumn group (4.55 ± 0.66), and the autumn group (4.55 ± 0.66) was lower than the winter group (5.16 ± 1.06). All differences were statistically significant (P < 0.05). Regarding ESR, the summer group (36.07 ± 30.74) was lower than the autumn group (63.07 ± 28.71) and the winter group (72.07 ± 41.44), with all differences being statistically significant (P < 0.05). For CRP, the summer group (19.17 ± 23.81) was lower than the winter group (60.54 ± 52.29), with statistically significant differences (P < 0.05). For rheumatoid factor (RF) and anti-citrullinated protein antibodies (ACPA), no statistically significant differences were observed among the four seasons (P > 0.05) (**Table 6**).

**Table 6:** Clinical characteristics of the patients.

Clinical characteristics		C1	C2	C3	C4	χ²/F	P
		(n=15)	(n=15)	(n=15)	(n=15)		
Sex, n(%)	Male	2(13.33)	2(13.33)	3(20.00)	3(20.00)	0.480	0.923
	Female	13(86.67)	13(86.67)	12(80.00)	12(80.00)
Age, years, mean(SD)		62.20(13.49)	55.40(9.68)	51.53(13.22)	54.20(12.38)	2.050	0.117
Duration, months, mean(SD)		77.73(80.71)	76.13(80.28)	80.00(73.27)	95.07(107.00)	0.149	0.930
BMI, kg/m², mean(SD)		20.44(3.95)	23.78(2.67)	23.48(3.31)	23.60(6.00)	2.196	0.099
DAS28-CRP, scores, mean(SD)		5.47(0.51)^xy^	4.82(0.79)	4.55(0.66)^z^	5.16(1.06)	3.910	0.013
ESR, mm/h, mean(SD)		56.53(28.00)	36.07(30.74)^yz^	63.07(28.71)	72.07(41.44)	3.288	0.027
CRP, mg/L, mean(SD)		42.38(37.05)	19.17(23.81)^z^	28.15(30.30)	60.54(52.29)	3.498	0.021
RF, RU/mL, mean(SD)		233.36(144.64)	166.12(172.86)	191.52(181.53)	303.76(152.13)	2.031	0.120
ACPA, RU/mL, mean(SD)		304.95(163.62)	227.74(193.45)	282.28(179.89)	317.32(157.69)	0.776	0.512

x compared with the summer group, P<0.05; y compared with the autumn group, P<0.05; z compared with the winter group, P<0.05.

### 3.3 Sample quality control

**Figure 2:**
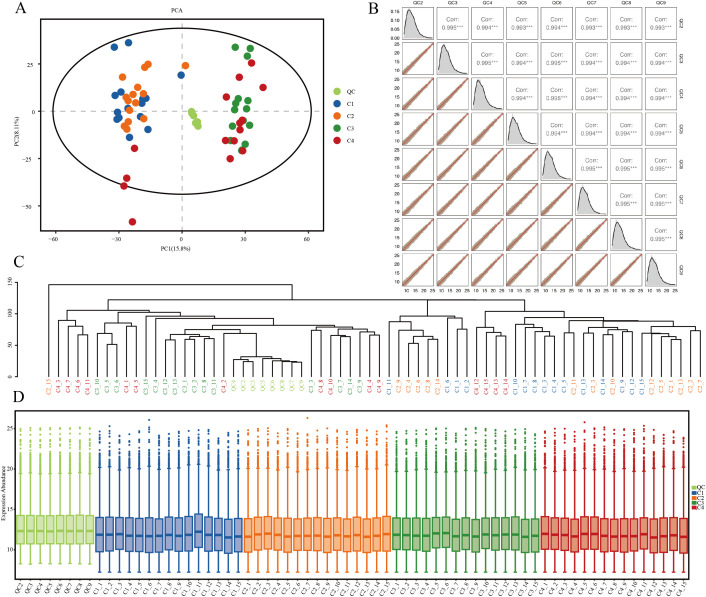
**(A)** PCA diagram of QC samples. **(B)** Correlation analysis diagram of QC samples. **(C)** Treeplot diagram of samples. **(D)** Boxplot diagram of samples.

PCA is an unsupervised data analysis method that uses an orthogonal transformation to convert the original random vectors, which are correlated in their components, into new random vectors with uncorrelated components. This transformation aims to preserve as much of the original variable information as possible, thereby achieving the goal of dimensionality reduction. After seven rounds of cross-validation, the PCA plot (**Figure 2A**) was obtained, showing that the QC samples are closely clustered together, indicating good experimental stability and reproducibility. To more intuitively demonstrate the correlation between QC samples, correlation analysis and plotting were performed on the QC samples (**Figure 2B**), showing that the correlation between QC samples is relatively close. Then, the Euclidean distances between samples were calculated based on the data matrix, and hierarchical clustering was performed on the sample distance matrix to plot the hierarchical clustering dendrogram of sample Euclidean distances (**Figure 2C**), where each branch end represents a sample, and samples clustered within the same branch are considered to have similar or close expression characteristics. The metabolite intensity boxplots of the samples were analyzed (**Figure 2D**). The height of the box reflects the degree of data variability to some extent; a flatter box indicates more concentrated data, and a shorter box also indicates more concentrated data. The further the median deviates from the center position of the upper and lower quartiles, the stronger the skewness of the distribution. It can be seen that the QC samples exhibit strong consistency and intra-group reproducibility.

### 3.4 Multivariate statistical analysis

**Figure 3:**
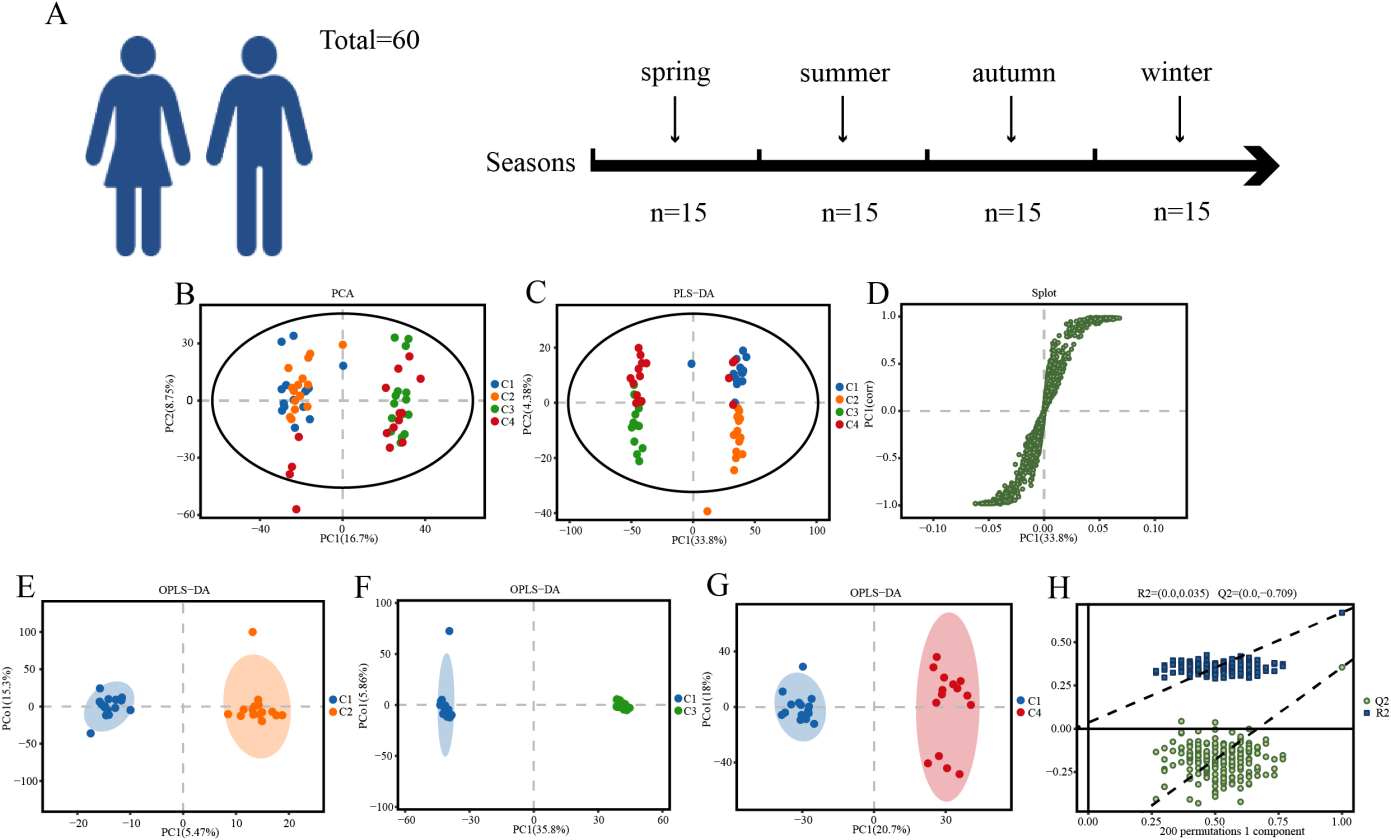
**(A)** Sample grouping diagram. **(B)** PLS-DA diagram. **(C)** PLS-DA diagram. **(D)** PLS-DA-Splot diagram. **(E–G)** OPLS-DA diagram. **(H)** Replacement test diagram.

Multivariate statistical analysis was performed on 60 samples (4 groups, 15 samples per group) (**Figure 3A**). First, unsupervised PCA was used to observe the overall distribution of the samples and the stability of the entire analysis process. Then, PLS-DA and OPLS-DA were used to distinguish the overall differences in metabolic profiles between groups and identify the differentially expressed metabolites between groups.

The PCA plot of the samples is shown in **Figure 3B** (where the horizontal axis PC1 represents the first principal component explanation rate, the vertical axis PC2 represents the second principal component explanation rate, each point in the figure represents a sample, different color-shape combinations indicate different sample groups, and the elliptical region represents the 95% confidence interval). It can be seen that nearly all samples from the four groups are within the 95% confidence interval. The data points are significantly distinguished in the PCA scatter plot. After seven rounds of cross-validation, the data from the four groups show strong consistency, indicating high reproducibility and reliability of the data. PLS-DA is a supervised discriminant statistical method that uses partial least squares regression to establish a relationship model between metabolite expression levels and sample groups to predict sample categories. By incorporating group variables, PLS-DA addresses the limitations of PCA. The PLS-DA model can explain and predict differences between two groups of samples, indicating good predictive capability (**Figure 3C**). In the PLS-DA-Splot plot, the x-axis represents the eigenvalues of metabolites affecting the comparison group, and the y-axis represents the correlation between sample scores and metabolites. Since eigenvalues and correlations are positively or negatively correlated, all points in the visualization plot are distributed in the first and third quadrants. Metabolites closer to the upper right and lower left corners indicate more significant differences (**Figure 3D**).

OPLS-DA is a supervised discriminant analysis statistical method. Based on the OPLS-DA model, the VIP values of each metabolite are obtained. It is a modification of PLS-DA, filtering out noise unrelated to classification information to enhance the model’s analytical capability and effectiveness, thereby maximizing the distinction between different groups within the model. Significant differences among the four groups are evident in the OPLS-DA score plot (**Figures 3E–G**). To prevent model overfitting, seven-fold cross-validation and RPT methods were employed to assess model quality. Typically, the closer the slopes of the R²Y and Q²Y lines approach the horizontal line, the more likely the model is to be overfitted. No overfitting was observed in any of the four groups (**Figure 3H**).

### 3.5 Differential comparison analysis

#### 3.5.1 Metabolite quantity information

**Figure 4:**
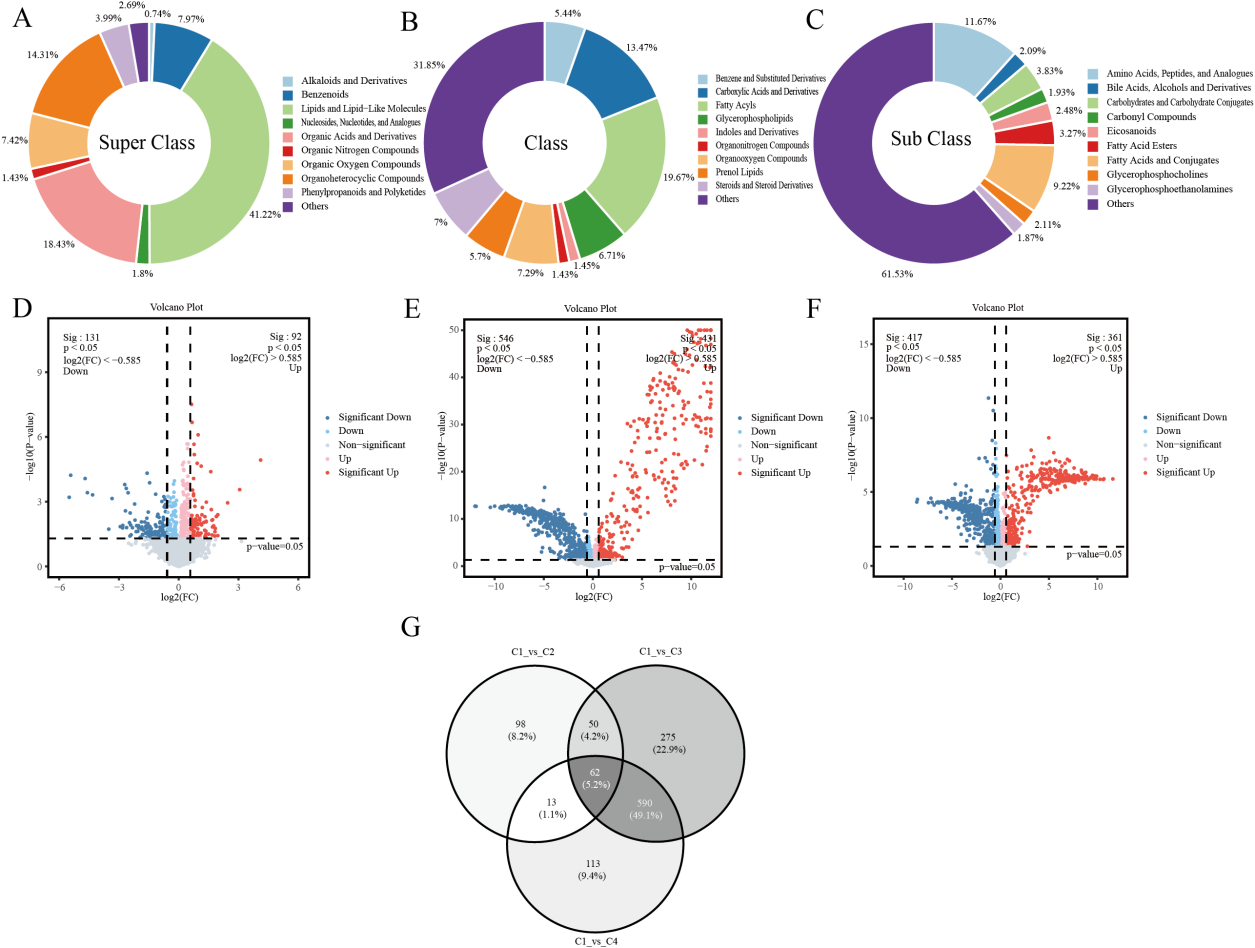
**(A)** Secondary classification of metabolites. **(B)** Tertiary classification of metabolites. **(C)** Quaternary classification of metabolites. **(D)** Volcano plot (C1-vs-C2). **(E)** Volcano plot (C1-vs-C3). **(F)** Volcano plot (C1-vs-C4). **(G)** Venn diagram.

A total of 3,787 metabolites were detected in the serum of four groups of RA patients. From the secondary classification of metabolites (**Figure 4A**), the top three categories were Lipids and Lipid-Like Molecules (41.22%), Organic Acids and Derivatives (18.43%), and Organoheterocyclic Compounds (14.31%); From the tertiary classification of metabolites (**Figure 4B**), the top three categories were Others (other compounds) (31.85%), Fatty Acyls (19.67%), and Carboxylic Acids and Derivatives (13.47%); From the perspective of the fourth-level classification of metabolites (**Figure 4C**), the top three categories by proportion are Others (other compounds) (61.53%), Amino Acids, Peptides, and Analogues (11.67%), and Fatty Acids and Conjugates (9.22%). Screen for differentially expressed metabolites in each group (screening criteria: p < 0.05, FC (fold change) ≥ 1.5, or FC ≤ 1/1.5), then display the top 10 metabolites with the smallest P-values in each group as shown in **Table 7**. As shown, in C1-vs-C2, the majority of metabolites belong to the categories “lipids and lipid-like molecules” and “organic acids and their derivatives,” and are predominantly up-regulated metabolites. In the C1-vs-C3 comparison, half of the metabolites belong to the “lipids and lipoid molecules” category, and all are up-regulated metabolites. In the C1-vs-C4 comparison, metabolites classified as “lipids and lipoid molecules” and those related to organic compounds account for a higher proportion, with both up-regulated and down-regulated metabolites present.

**Table 7:** Information on the top 10 metabolites with the smallest P-values in each comparison group.

Group	Number	Metabolites	Super class	Class	Sub class	VIP	Regulation
	1	Hexan-1-ol	Lipids and lipid-like molecules	Fatty Acyls	Fatty alcohols	2.17	Up
	2	N-(9-Oxodecyl)acetamide	Organic acids and derivatives	Carboxylic acids and derivatives	Carboxylic acid derivatives	2.29	Up
	3	N-ISOPROPYLACRYLAMIDE	Organic acids and derivatives	Carboxylic acids and derivatives	Acrylic acids and derivatives	2.66	Up
	4	Behenic acid(d3)	Lipids and lipid-like molecules	Fatty Acyls	Fatty acids and conjugates	2.29	Up
C1-vs-C2	5	Alanylserine	Organic acids and derivatives	Carboxylic acids and derivatives	Amino acids, peptides, and analogues	2.31	Up
	6	4-Methyl-umbelliferyl-N-acetyl-chitobiose	Phenylpropanoids and polyketides	Coumarins and derivatives	Coumarin glycosides	5.16	Up
	7	8,8-Dimethoxy-2,6-dimethyl-2-octanol	Organic oxygen compounds	Organooxygen compounds	Alcohols and polyols	2.48	Up
	8	5-hydroxypropafenone	Phenylpropanoids and polyketides	Linear 1,3-diarylpropanoids	Cinnamylphenols	2.75	Up
	9	MG(0:0/18:2+=O/0:0)	Lipids and lipid-like molecules	Fatty Acyls	Lineolic acids and derivatives	3.19	Up
	10	PE(0:0/20:4)	Lipids and lipid-like molecules	Glycerophospholipids	Glycerophosphoethanolamines	3.10	Down
	1	PS(12:0/20:4)	Lipids and lipid-like molecules	Glycerophospholipids	Glycerophosphoserines	3.74	Up
	2	N-Arachidonoyl Phenylalanine	Organic acids and derivatives	Carboxylic acids and derivatives	Amino acids, peptides, and analogues	3.48	Up
	3	Uriolide	Lipids and lipid-like molecules	Prenol lipids	Triterpenoids	3.77	Up
	4	Lamivudine-triphosphate	Nucleosides, nucleotides, and analogues	Nucleoside and nucleotide analogues	Unclassified	3.58	Up
C1-vs-C3	5	Ceftriaxone	Organoheterocyclic compounds	Lactams	Beta lactams	3.45	Up
	6	PS(17:0/12:0)	Lipids and lipid-like molecules	Glycerophospholipids	Glycerophosphoserines	3.54	Up
	7	1-(2-methoxy-5Z,9Z-hexacosadienyl)-sn-glycero-3-phosphoserine	Lipids and lipid-like molecules	Glycerophospholipids	Glycerophosphoserines	3.66	Up
	8	Stavudine triphosphate	Nucleosides, nucleotides, and analogues	Pyrimidine nucleotides	Pyrimidine deoxyribonucleotides	3.26	Up
	9	PE-Cer(d14:2/20:1)	Lipids and lipid-like molecules	Sphingolipids	Phosphosphingolipids	3.75	Up
	10	Perfluorodecalin	Organohalogen compounds	Organofluorides	Unclassified	3.30	Up
	1	Gabapentin	Organic acids and derivatives	Carboxylic acids and derivatives	Amino acids, peptides, and analogues	1.44	Down
	2	9,10-DiHOME	Lipids and lipid-like molecules	Fatty Acyls	Fatty acids and conjugates	1.14	Down
	3	4-Methyl-umbelliferyl-N-acetyl-chitobiose	Phenylpropanoids and polyketides	Coumarins and derivatives	Coumarin glycosides	2.79	Up
	4	7-Keto-8-aminopelargonic acid	Lipids and lipid-like molecules	Fatty Acyls	Fatty acids and conjugates	1.16	Down
C1-vs-C4	5	Alanine amine	Organic acids and derivatives	Carboxylic acids and derivatives	Amino acids, peptides, and analogues	2.24	Up
	6	Perfluorooctane sulfonamidoacetic acid	Organohalogen compounds	Alkyl halides	Alkyl fluorides	2.96	Up
	7	1-methyl-cyclopentanol	Organic oxygen compounds	Organooxygen compounds	Alcohols and polyols	0.95	Up
	8	PA(i-24:0/PGJ2)	Lipids and lipid-like molecules	Fatty Acyls	Eicosanoids	2.31	Up
	9	xi-2-Hexyl-5-methyl-3(2H)-furanone	Organoheterocyclic compounds	Dihydrofurans	Furanones	1.60	Down
	10	PA(O-20:0/17:1)	Lipids and lipid-like molecules	Glycerophospholipids	Glycerophosphates	1.86	Down

Using a volcano plot to visualize p-values and fold change (FC) values facilitates the screening of differentially expressed metabolites, with red and blue dots indicating significantly different metabolites after screening. A total of 223 significantly different metabolites were identified between the C1-vs-C2 groups, including 92 up-regulated and 131 down-regulated metabolites (**Figure 4D**). A total of 977 significantly different metabolites were screened and identified between the C1-vs-C3 groups, including 431 upregulated and 546 downregulated metabolites (**Figure 4E**). A total of 778 significantly different metabolites were screened and identified between the C1-vs-C4 groups, including 361 upregulated and 417 downregulated metabolites (**Figure 4F**). Each point in the figure represents a metabolite, with the x-axis showing the log2 (FC) values for the two groups, and the y-axis showing the -log10 (p-value) values. Red points indicate significantly up-regulated differential metabolites (p < 0.05, and FC > 1), blue points indicate significantly downregulated differential metabolites (p < 0.05 and FC < 1), and gray points represent metabolites with no significant differences. The Venn diagram (**Figure 4G**) shows that there are 62 common differential metabolites among the three comparison groups, with the top 20 metabolites displayed in **Table 8** based on the smallest p-value.

**Table 8:** Information on the top 20 common differentially expressed metabolites.

Number	Metabolites	Super class	Class	Sub class
1	Hexan-1-ol	Lipids and lipid-like molecules	Fatty Acyls	Fatty alcohols
2	N-(9-Oxodecyl)acetamide	Organic acids and derivatives	Carboxylic acids and derivatives	Carboxylic acid derivatives
3	4-Methyl-umbelliferyl-N-acetyl-chitobiose	Phenylpropanoids and polyketides	Coumarins and derivatives	Coumarin glycosides
4	8,8-Dimethoxy-2,6-dimethyl-2-octanol	Organic oxygen compounds	Organooxygen compounds	Alcohols and polyols
5	MG(0:0/18:2+=O/0:0)	Lipids and lipid-like molecules	Fatty Acyls	Lineolic acids and derivatives
6	PE(0:0/20:4)	Lipids and lipid-like molecules	Glycerophospholipids	Glycerophosphoethanolamines
7	PC(O-16:0/22:5)	Lipids and lipid-like molecules	Glycerophospholipids	Glycerophosphocholines
8	Stizolamine	Organoheterocyclic compounds	Diazines	Pyrazines
9	PS(21:0/22:6)	Lipids and lipid-like molecules	Glycerophospholipids	Glycerophosphoserines
10	28:7(n-6)	Lipids and lipid-like molecules	Fatty Acyls	Fatty acids and conjugates
11	PC(O-16:0/18:2)	Lipids and lipid-like molecules	Glycerophospholipids	Glycerophosphocholines
12	3,5-Dihydroxytetradecanoylcarnitine	Lipids and lipid-like molecules	Fatty Acyls	Fatty acid esters
13	Ilomastat	Organic acids and derivatives	Carboxylic acids and derivatives	Amino acids, peptides, and analogues
14	PC(P-16:0/22:4)	Lipids and lipid-like molecules	Glycerophospholipids	Glycerophosphocholines
15	PE(18:2/18:0)	Lipids and lipid-like molecules	Glycerophospholipids	Glycerophosphoethanolamines
16	1,4-bis[(2-ethylhexyl)oxy]-1,4-dioxobutane-2-sulfonic acid	Lipids and lipid-like molecules	Fatty Acyls	Fatty acid esters
17	PC(O-16:0/20:4)	Lipids and lipid-like molecules	Glycerophospholipids	Glycerophosphocholines
18	2,2’-(3-methylcyclohexane-1,1-diyl)diacetic acid	Organic acids and derivatives	Carboxylic acids and derivatives	Dicarboxylic acids and derivatives
19	PE(22:4/19:0)	Lipids and lipid-like molecules	Glycerophospholipids	Glycerophosphoethanolamines
20	Cyanidin 7-arabinoside	Phenylpropanoids and polyketides	Flavonoids	Hydroxyflavonoids

#### 3.5.2 Analysis of important differential metabolites

**Figure 5:**
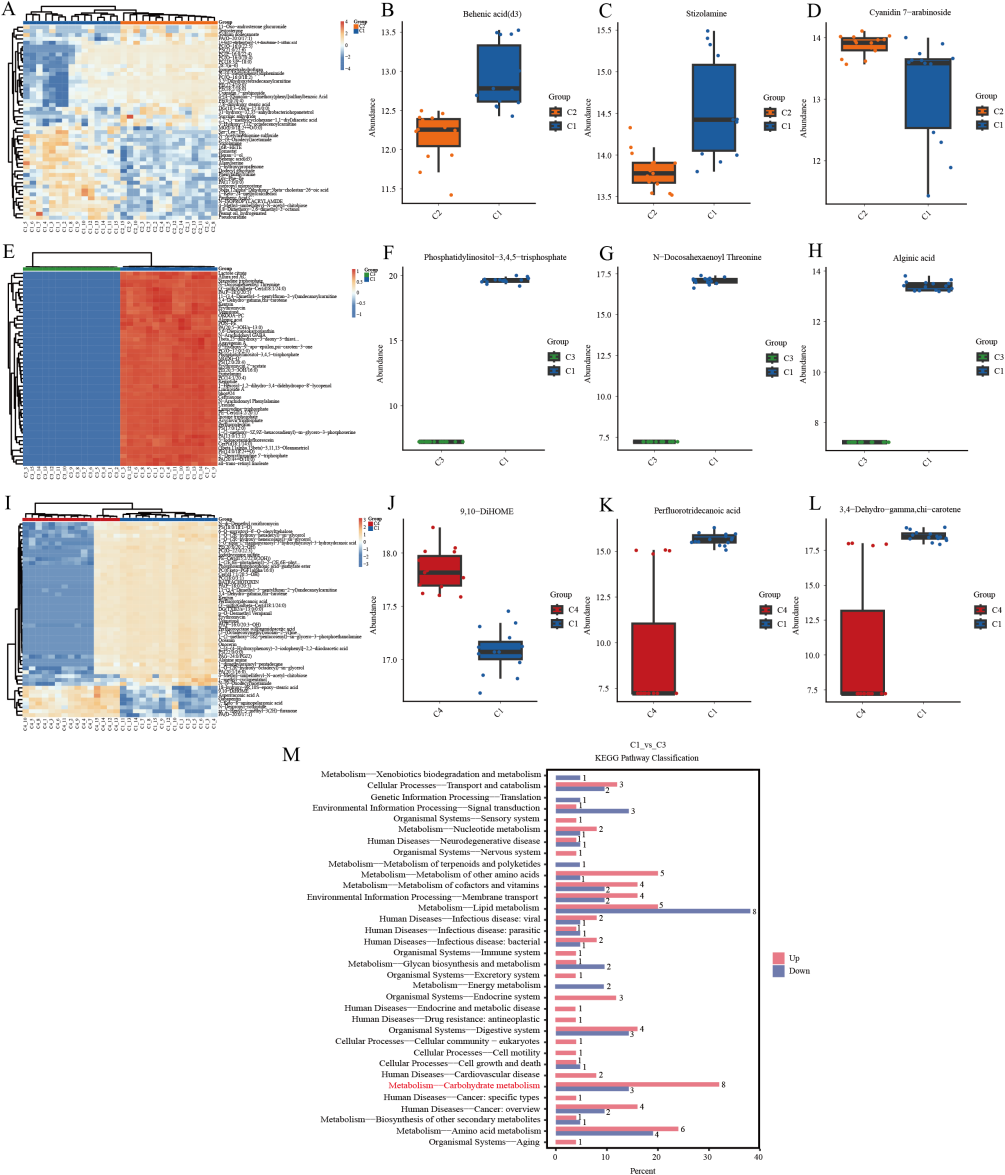
**(A)** C1-vs-C2 clustering heat map. **(B–D)** C1-vs-C2 significantly different metabolites. **(E)** C1-vs-C3 clustering heatmap. **(F–H)** C1-vs-C3 significantly different metabolites. **(I)** C1-vs-C4 clustering heatmap. **(J–L)** C1-vs-C4 significantly different metabolites. **(M)** KEGG enrichment analysis of C1-vs-C3 significantly different metabolites.

To more intuitively illustrate the relationships between samples and the expression differences of metabolites across different samples, hierarchical clustering was performed on all significantly different metabolites and on the top 50 significantly different metabolites ranked by p-value. The x-axis represents sample names, the y-axis represents different metabolites, and colors ranging from blue to red indicate metabolite expression abundance from low to high. It is evident that the expression abundance of metabolites varies significantly across groups, with notable differences within each group (**Figures 5A, E, I**). The key differential metabolites in C1-vs-C2 include Behenic acid (d3), Stizolamine, and Cyanidin 7-arabinoside (**Figures 5B–D**). Behenic acid (d3) and Stizolamine are highly abundant in C1, while Cyanidin 7-arabinoside is highly abundant in C2. C1-vs-C3 key differential metabolites include Phosphatidylinositol-3,4,5-trisphosphate, N-Docosahexaenoyl Threonine, and Alginic acid (**Figures 5F–H**), all of which are highly abundant in C1. C1-vs-C4 important differential metabolites include 9,10-DiHOME, perfluorotridecanoic acid, and 3,4-dehydro-gamma,chi-carotene (**Figures 5J–L**), with 9,10-DiHOME being highly abundant in C4, and perfluorotridecanoic acid and 3,4-dehydro-gamma,chi-carotene being highly abundant in C1. KEGG enrichment analysis (**Figure 5M**) showed that alginic acid was enriched in the carbohydrate metabolism pathway, which enriched 11 differential metabolites, with 8 up-regulated and 3 down-regulated.

## 4 Discussion

### 4.1 Seasonal patterns in the serum metabolome of RA patients and the biological significance of core differentially expressed metabolites

We conducted a systematic analysis of seasonal differences in serum metabolomes of 60 RA patients using LC-MS/MS technology, and for the first time identified distinct metabolic profiles across different seasons in the RA population in Guiyang. This provides evidence for understanding the regulatory mechanisms of seasonal factors on the pathophysiological processes of RA. This study employs a cross-sectional design, analyzing population-level metabolic differences grouped by season. The conclusions reflect metabolic associations between RA patient cohorts across different seasons, rather than dynamic trajectories within individuals over seasonal changes. Inherent inter-individual heterogeneity in genetic background and lifestyle habits may influence metabolic phenotypes. To mitigate this, we controlled for confounding factors such as gender, age, BMI, and dietary patterns through stringent inclusion and exclusion criteria (**Table 6** shows no statistically significant differences in these metrics across the four groups). Additionally, PCA analysis validated the clustering of samples within groups (**Figure 3B**), thereby reducing interference from individual heterogeneity. Longitudinal follow-up studies can track the metabolic dynamics of the same patient across different seasons, further validating the seasonal associations identified in this study. This will be a key focus of our subsequent research. The study results showed no statistically significant differences among the four patient groups in terms of gender, age, disease duration, BMI, RF, or ACPA. The male-to-female ratio of 1:5 observed in this study was not artificially set but rather the natural outcome of strictly adhering to the inclusion criteria for random case recruitment. Epidemiological data indicate a significant gender disparity in RA incidence, with women exhibiting markedly higher rates than men (approximately 2–3 times higher globally) (20). The gender ratio in this study aligns with the natural distribution observed in clinical RA patient populations, demonstrating strong clinical representativeness. Regarding DAS28-CRP scores, the spring group (5.47 ± 0.51) was higher than the summer group (4.82 ± 0.79) and the spring group (4.55 ± 0.66) was higher than the winter group (5.16 ± 0.51). the spring group (5.47 ± 0.51) was higher than the autumn group (4.55 ± 0.66), and the autumn group (4.55 ± 0.66) was lower than the winter group (5.16 ± 1.06). The spring group had the highest score throughout the year, while the autumn group had the lowest. Clinically, the scoring patterns aligned with seasonal fluctuations in joint swelling counts, tender points, and morning stiffness duration. Patients with higher spring scores exhibited marked joint swelling and tenderness, significantly exceeding autumn levels. This seasonal variation further confirms the influence of seasonal factors on RA inflammatory activity. Clinically, this suggests enhanced monitoring frequency of inflammatory markers during spring, while autumn may warrant optimizing maintenance therapy regimens considering lower disease activity. The study results showed that a total of 3,787 metabolites were detected in the serum of the four patient groups. In the secondary classification, Lipids and Lipid-Like Molecules accounted for 41.22%, Fatty Acyls accounted for 19.67% in the tertiary classification, and Fatty Acids and Conjugates accounted for 9.22% in the quaternary classification. As shown in **Table 7**, the classification of differential metabolites was also primarily “Lipids and Lipid-Like Molecules”. This indicates a close association between lipid metabolism and RA. However, due to the lack of parallel control data from healthy individuals or those with non-RA inflammatory diseases, it remains unclear whether this association is specific to RA. Studies have found that RA patients exhibit abnormal lipid profiles, with untreated or active RA patients showing reduced high-density lipoprotein cholesterol (HDL-C) and LDL-C levels (21). HDL-C is negatively correlated with C-reactive protein (CRP), while the TC/HDL-C and LDL/HDL ratios are positively correlated with CRP (22). Based on existing research reports, lipids may be involved in the inflammatory process of RA (with a particular focus on the association between lipids and macrophages). As membrane components, the lipid composition of cell membranes can alter membrane fluidity or lipid raft structure, thereby influencing cellular signaling pathways (23). In the absence of fatty acid synthase, changes in cholesterol content and lipid raft composition can reduce macrophage inflammation (24). Additionally, existing research suggests that exogenous lipids such as long-chain saturated fatty acids like palmitic acid or stearic acid may directly serve as pro-inflammatory signals (25). Lipids may also influence fibroblast-like synovial cell (FLS) function through multiple pathways. For instance, free fatty acids (FA) may promote the formation of a pro-inflammatory environment in RA FLS and increase pro-inflammatory cytokine and chemokine secretion in a dose-dependent manner (with similar pro-inflammatory effects observed for both saturated and unsaturated free fatty acids) (26). However, the aforementioned association between lipids and FLS function remains speculative based on existing literature. This study did not measure cytokine profiles or FLS activity, and whether this represents a key pathway in RA inflammation regulation requires targeted functional validation. Certain antirheumatic drugs, such as leflunomide, can improve dyslipidemia, reduce lipid content in the liver, and improve disrupted glucose and lipid metabolism in the body. RNA sequencing results show that leflunomide effectively regulates gene expression related to lipid metabolism pathways (27). Additionally, a plasma lipidomics study found that methotrexate treatment for RA alters lipid levels and quantified these changes (28). Zheng DC et al. found that SR9243 regulates glycolytic metabolism, thereby inhibiting M1 macrophage polarization and activation, alleviating adjuvant-induced RA, and preventing bone erosion (29). These studies suggest that anti-rheumatic drugs may exert their therapeutic effects by regulating lipid metabolism or related metabolic pathways. However, this study did not verify a direct association between lipid metabolism and the drugs’ mechanisms of action. This hypothesis requires further experimental validation through studies examining the relationship between drug intervention and lipid metabolism, as well as immune cell function.

We found that the C1-vs-C2, C1-vs-C3, and C1-vs-C4 groups all contained multiple differentially expressed metabolites. Among them, the key differentially expressed metabolites in the C1-vs-C2 group included Behenic acid (d3), Stizolamine, and Cyanidin 7-arabinoside. Behenic acid (d3) and Stizolamine were highly abundant in C1, while Cyanidin 7-arabinoside is more abundant in C2. Research suggests that Behenic acid may be used as a dietary supplement for the prevention and treatment of diseases. It reduces inflammation and insulin resistance in pregnant diabetic mice by inhibiting the activation of the TLR4/NF-κB signaling pathway (30). Supplementing with Behenic acid may serve as a dietary therapy for type 2 diabetes (31). Stizolamine is a naturally occurring non-protein amino acid found in the seeds of the legume Stizolobium deeringianum. Stizolamine and its metabolic derivatives are potential ligands for the aryl hydrocarbon receptor (AhR), which plays a key role in self-antigen tolerance. Stizolamine may serve as a potential dietary strategy for managing autoimmune diseases (32). Cyanidin 7-arabinoside is a naturally occurring anthocyanin flavonoid compound, which is hypothesized to possess the bioactive properties of anthocyanins. Anthocyanins are water-soluble pigments widely found in plants, exhibiting various bioactive functions such as antioxidant and anti-inflammatory effects (33). Anthocyanins can alleviate autoimmune arthritis by inhibiting the development of Th17 cells and the synthesis of pro-inflammatory cytokines, while also reducing oxidative stress levels (34). Key differential metabolites between C1 and C3 include phosphatidylinositol-3,4,5-trisphosphate, N-docosahexaenoyl threonine, and alginic acid, all of which are highly abundant in C1. Phosphatidylinositol-3,4,5-trisphosphate (PIP3), as a crucial phospholipid second messenger, has been shown to participate in immune cell activation, inflammatory cytokine release, and maintenance of immune tolerance by regulating intracellular signaling pathways. Abnormal signaling may be associated with imbalanced inflammatory responses and the development of autoimmune diseases (35, 36). Moreover, targeting PIP3 signaling may suppress macrophage immune function (considered a potential therapeutic target for inflammation-related diseases) (37). However, this study did not assess immune cell activation status or expression of key molecules in the PIP3 signaling pathway. Its specific role in seasonal inflammatory fluctuations of RA remains a hypothetical exploration requiring further experimental validation. N-Docosahexaenoyl Threonine is an endogenous lipoic acid formed by the amide bond between docosahexaenoic acid (DHA) and threonine. As a derivative of DHA, it may inherit some of DHA’s biological activities (such as anti-inflammatory effects). Research has shown that after DHA inserts into the non-lipid rafts region of the cell membrane, it “squeezes” sphingomyelin and cholesterol molecules into larger lipid raft aggregates. This change in lipid raft structure may act like a switch, blocking inflammatory signal transmission within cells and thereby exerting an anti-inflammatory effect (38). Supplementing with DHA can reduce inflammation in RA, and DHA and its derivatives have become potential therapeutic agents for RA (39, 40). Based on the aforementioned literature, N-Docosahexaenoyl Threonine may participate in RA inflammation regulation through a similar mechanism. However, this study did not validate its anti-inflammatory activity or signaling pathway mechanisms. This association requires further confirmation through subsequent cellular experiments or animal models. Alginic acid is a natural linear polysaccharide found in brown algae and other seaweeds, which possesses anti-inflammatory potential and can alleviate arthritis induced by type II collagen in experimental animals (41). Foods or medications containing alginic acid may potentially be used to treat RA. However, this study did not measure inflammatory factor levels or the activity of related anti-inflammatory signaling pathways. Whether alginic acid influences the progression of RA by regulating lipid metabolism requires further targeted experimental validation.

In the C1-vs-C3 comparison, N-Arachidonoyl Phenylalanine was upregulated, indicating that N-Arachidonoyl Phenylalanine levels were higher in autumn than in spring. N-Arachidonoyl Phenylalanine is a derivative of arachidonic acid and may influence inflammatory responses by participating in arachidonic acid-related signaling pathways. Research has shown that N-Arachidonoyl Phenylalanine is an N-arachidonoyl amino acid derivative with good inhibitory effects on phospholipase D, which catalyzes the hydrolysis of phospholipids to produce phospholipids and active alcohols, thereby participating in the biosynthesis of lipid signaling molecules (42). Moreover, N-arachidonoyl-serotonin from the same family exhibits both anti-inflammatory and anti-hypersensitivity effects, offering advantages in therapeutic efficacy and reduced adverse reactions (43). Lawton SK et al. found that N-Arachidonoyl Dopamine (NADA), as an endogenous lipid, significantly reduces systemic inflammatory responses in a lipopolysaccharide (LPS)-induced inflammatory mouse model. NADA exhibits novel transient receptor potential vanilloid 1 (TRPV1)-dependent anti-inflammatory properties, and suggests that the endovanilloid system may serve as a therapeutic target in acute inflammation (44). Therefore, we speculate that N-Arachidonoyl Phenylalanine may possess certain anti-inflammatory effects, and the increase in N-Arachidonoyl Phenylalanine in autumn may be one of the reasons why RA disease activity is lower in autumn than in spring. However, this study did not directly assess the effects of this metabolite on immune cell function or inflammatory factors. This hypothesis requires further validation through *in vitro* anti-inflammatory experiments and *in vivo* correlation studies. Upregulation of PA (i-24:0/PGJ2) in C1-vs-C4 indicates that winter PA (i-24:0/PGJ2) levels are higher than spring levels. PGJ2 (prostaglandin J2) is a type of prostaglandin, which are important inflammatory mediators that can cause symptoms such as pain, fever, and swelling at the site of inflammation, and participate in the regulation of the inflammatory process. Studies have shown that prostaglandin E2, a potent lipid mediator generated from arachidonic acid (AA) via cyclooxygenase (COX), plays a significant role in the pathogenesis of RA (45). Inhibiting the release of inflammatory mediators such as prostaglandin E2 (PGE_2_) through medication can alleviate RA symptoms (46). Based on this speculation, PA (i-24:0/PGJ2) may participate in RA inflammatory regulation through a similar mechanism. However, since this study did not examine prostaglandin-related signaling pathways or inflammatory mediator levels, its role in seasonal metabolic fluctuations of RA requires further experimental validation. As shown in **Table 7**, the “Fatty Acyls” and “Glycerophospholipids” categories account for a significant proportion of the top 20 common differential metabolites, confirming the important role of lipid metabolism in the seasonal fluctuations of RA. Fatty acyls are closely associated with inflammation, oxidative damage, and cellular signaling processes (47, 48). Intervening in biological processes related to fatty acyls may have positive effects on inflammation and oxidative damage. Glycerophospholipids are major components of biological membranes and key molecules in cellular signaling. Their metabolism is closely associated with inflammatory responses and immune regulation, influencing the body’s inflammatory state and immune homeostasis through various mechanisms, including serving as precursors to inflammatory mediators, regulating immune cell activation, and participating in immune signaling. Glycerophospholipids include phosphatidylcholine (PC) and phosphatidylserine (PS), among others. PC is the most abundant glycerophospholipid in biological membranes and serves as the primary storage reservoir for fatty acids (especially AA). When cells are exposed to inflammatory stimuli (such as bacterial LPS or cytokines), phospholipase A_2_ (PLA_2_) is activated, hydrolyzing PC to release AA; AA is further metabolized by COX-1/2 into prostaglandins (e.g., PGE_2_, which induces vasodilation and pain) or by lipoxygenase (LOX) into leukotrienes (e.g., LTB_4_, which recruits neutrophil infiltration). These molecules are core mediators of inflammation (49–51). In normal cells, PS is located on the inner side of the cell membrane. During apoptosis, PS is flipped to the membrane surface, serving as a “phagocytic signal” recognized by PS receptors on macrophages, initiating the clearance of apoptotic cells. If PS flipping is abnormal or phagocytic function is defective, accumulated apoptotic cells release pro-inflammatory substances, triggering chronic inflammation (such as autoimmune diseases like RA) (52, 53). Thus, glycerophospholipids play a critical role in the initiation, progression, resolution, and maintenance of immune homeostasis. Disruption of their metabolic balance can lead to uncontrolled inflammation or immune dysfunction, and targeting glycerophospholipid metabolism (such as inhibiting PLA_2_ or regulating PS signaling) may represent a potential therapeutic strategy for inflammatory and immune diseases (54, 55). However, this study did not validate PLA_2_ activity, PS eversion status, or levels of related inflammatory mediators in RA patients. The applicability of the aforementioned mechanisms to seasonal metabolic fluctuations in RA requires further experimental confirmation.

Key metabolic differences between C1 and C4 include 9,10-DiHOME, perfluorotridecanoic acid, and 3,4-dehydro-gamma,chi-carotene. 9,10-DiHOME is more abundant in C4, while perfluorotridecanoic acid and 3,4-dehydro-gamma,chi-carotene are more abundant in C1. 9,10-DiHOME is negatively correlated with body mass index. It improves systemic metabolism by activating brown adipose tissue and may serve as a potential therapeutic target for metabolic syndrome (56). 9,10-DiHOME is a bioactive lipid with Treg-inducing activity and may act as a potential biomarker for colitis (57). Perfluorotridecanoic acid is a typical perfluorinated and polyfluorinated alkyl substance that exhibits toxic effects on the body (including inflammation and immune dysregulation), leading to increased levels of interleukin-6 (IL-6) in the body (58). It can inhibit the differentiation of fetal interstitial cells in male fetuses by increasing oxidative stress and inducing autophagy (59). However, this study did not measure IL-6 levels or oxidative stress status in RA patients, and the specific regulatory role of this substance in RA remains to be verified. 3,4-Dehydro-gamma,chi-carotene is a naturally occurring carotenoid belonging to the carotenoid family. Direct studies on 3,4-Dehydro-gamma,chi-carotene are currently limited, but based on its chemical structural characteristics, it is speculated to share common mechanisms with other carotenoids. Carotenoids are a class of lipid-soluble pigments widely found in plants, algae, and certain microorganisms, and they are extensively studied for their antioxidant, anti-inflammatory, and immunomodulatory activities (60, 61). Carotenoids can provide neuroprotective effects by inhibiting neuroinflammation, microglia activation, excitotoxic pathways, regulating autophagy, reducing oxidative damage, and activating defensive antioxidant enzymes. They can act as both antioxidants and anti-inflammatory agents (62, 63). As a carotenoid derivative, 3,4-dehydro-gamma,chi-carotene may exhibit similar biological activities. However, this study did not evaluate its antioxidant and anti-inflammatory functions or related signaling pathways. This hypothesis requires confirmation through subsequent functional experiments.

KEGG enrichment analysis shows that alginic acid is enriched in the carbohydrate metabolism pathway, which contains 11 differentially expressed metabolites, 8 of which are upregulated and 3 of which are downregulated. The Carbohydrate Metabolism pathway is a biochemical reaction network within cells that involves the breakdown, utilization, and synthesis of carbohydrates (primarily glucose) into storage forms. Its core functions include providing energy to cells, maintaining stable blood sugar levels, and supplying precursor substances for the synthesis of other biomolecules (such as lipids and amino acids). It serves as a central hub in cellular metabolism, closely interconnected with lipid metabolism (glucose can be converted into fatty acids), amino acid metabolism (intermediate products of glucose can synthesize non-essential amino acids), and nucleotide metabolism (phosphoribose originates from the pentose phosphate pathway), collectively maintaining cellular material and energy balance. Additionally, the lipid metabolism pathway is enriched with 13 differentially expressed metabolites, with 5 upregulated and 8 downregulated, further confirming the crucial role of lipid metabolism in the onset and progression of RA. Based on the aforementioned metabolomics findings and existing literature, it is speculated that lipid metabolism may play a significant role in the onset and progression of RA. However, due to the lack of parallel control data from healthy individuals or those with non-RA inflammatory diseases, it remains unclear whether these lipid metabolic alterations represent RA-specific pathological phenotypes or non-specific manifestations common to inflammatory states or seasonal metabolic fluctuations. Their direct association with RA-related immune inflammation requires further validation.

### 4.2 Analysis of key confounding factors influencing seasonal fluctuations in RA patients’ metabolism

In metabolite expression analysis, we have considered and controlled for potential gender-related differences. By integrating literature-reported gender-specific metabolic pathways (such as estrogen-mediated pathways), we functionally annotated differential metabolites to minimize the impact of gender-specific metabolites on overall seasonal variation conclusions, ensuring the reliability of our findings. Furthermore, age and gender, as inherent physiological factors, interact with seasonal influences to further shape metabolic fluctuations in RA patients. Regarding age-related metabolic differences, elderly patients exhibit diminished mitochondrial function and reduced lipolytic capacity, resulting in weaker metabolic adaptation to seasonal temperature changes. Winter high-fat diets induce more pronounced elevations in LDL-C and glycerophospholipid metabolic disorders in this group. Core differential metabolites (e.g., PIP3, PA (i-24:0/PGJ2)) exhibit greater seasonal fluctuations than in younger patients. In contrast, younger patients demonstrate stronger metabolic plasticity, with seasonal metabolic differences primarily concentrated in energy metabolism pathways and exhibiting relatively smaller fluctuations. Seasonal fluctuations in serum metabolites among RA patients in this study may also be indirectly regulated by physical activity, sunlight exposure (vitamin D), and seasonal lifestyle variations. Regarding physical activity, Guiyang’s cold winters and hot summers lead to significantly reduced outdoor exercise in winter and increased activity frequency in summer. Winter sedentary behavior decreases energy expenditure, promoting lipid accumulation (e.g., triglycerides) and exacerbating lipid metabolism disorders. Regarding sunlight and vitamin D, shorter daylight hours and weaker intensity in winter lead to insufficient vitamin D synthesis. Vitamin D inhibits pro-inflammatory cytokine release and regulates lipid metabolism (64, 65). Winter vitamin D deficiency may exacerbate lipid metabolism disorders and inflammatory responses. In contrast, abundant summer sunlight increases vitamin D synthesis, regulating the expression of anti-inflammatory metabolites, which may mitigate metabolic fluctuations and inflammation. Lifestyle factors: Winter diets often emphasize high-fat, high-sugar foods, increasing the supply of lipid metabolism substrates. This leads to abnormal accumulation of metabolites like glycerophospholipids and fatty acids, intensifying inflammation. Summer diets feature increased vegetable and fruit consumption, delivering more anti-inflammatory compounds like anthocyanins and carotenoids that promote expression of metabolites such as cyanidin 7-arabinoside. These seasonal lifestyle variations synergize with metabolite fluctuations to jointly influence RA inflammatory activity.

Our data reveal distinct seasonal fluctuations in disease activity markers such as DAS28-CRP, ESR, and CRP: DAS28-CRP scores peaked in spring, significantly higher than in summer and autumn; Winter CRP levels are significantly higher than summer levels, and ESR also shows a trend of being higher in winter and autumn than in summer. Among the differential metabolites, Lipids and Lipid-Like Molecules account for 41.22%, representing the most predominant differential category. It is known that inflammatory responses and lipid metabolism exhibit a close bidirectional regulatory relationship. Inflammatory factors such as IL-6 can act as key regulators in metabolic networks (66), influencing lipid profiles, while lipid metabolites like long-chain saturated fatty acids and glycerophospholipid derivatives may also participate in inflammatory signaling (67). This suggests that the observed metabolomic differences in this study may indeed result from dual possibilities: a “direct effect of seasonal factors” and “indirect mediation by inflammatory burden.” Due to the cross-sectional design of this study, it is currently impossible to definitively distinguish the independent roles of these two factors. To minimize confounding effects, we employed several strategies: strictly enrolling patients not using hormones or immunosuppressants; selecting core metabolites with season-specific expression and RA-inflammatory biological functions; and validating cross-linkages between lipid metabolism and inflammatory pathways via KEGG pathway analysis. Our findings suggest that inflammatory burden likely serves as a potential intermediate mediator in seasonally regulated metabolic processes rather than an entirely independent confounder. This does not undermine the core conclusion that seasonal factors participate in regulating the RA metabolic-inflammatory network. In subsequent studies, we will further clarify the causal relationship between these two factors to enhance the reliability of our conclusions. This will be achieved by conducting longitudinal follow-up to track the dynamic changes in metabolites and inflammatory markers across different seasons in the same patients, supplementing *in vitro* functional experiments to validate the direct regulatory effects of core metabolites on macrophages and fibroblast-like synovial cells, and expanding the sample size. It is noteworthy that among the differentially expressed metabolites identified in this study, some belong to environmentally derived compounds (e.g., perfluorotridecanoic acid). These substances primarily originate from dietary intake and environmental exposure. Their seasonal fluctuations may reflect variations in environmental pollutant levels or dietary patterns across different seasons in Guiyang (e.g., potential perfluorinated compound residues in high-fat winter diets), rather than intrinsic pathophysiological changes associated with RA. Seasonal variations in exogenous compounds may inform environmental risk factor studies for RA, but caution is warranted when interpreting their direct association with disease pathophysiology. Furthermore, previous studies have demonstrated that commonly used disease-modifying antirheumatic drugs (DMARDs) such as methotrexate and leflunomide exert distinct regulatory effects on lipid metabolism. Therefore, during patient enrollment, we strictly adhered to inclusion criteria, excluding all patients with a history of relevant DMARD use.

## 5 Conclusion

This study is the first to reveal seasonal differences in serum metabolomics in RA patients in the Guiyang region using LC-MS/MS technology, suggesting that lipid metabolism may play a significant role in seasonal fluctuations of RA. The study found that the differential metabolites in RA patients across different seasons primarily consist of lipids and lipid analogues, such as Behenic acid (d3), Stizolamine, and Cyanidin 7-arabinoside. These metabolites may influence the progression of RA by regulating inflammatory pathways, immune cell function, and lipid metabolic balance. KEGG enrichment analysis further validates that lipid metabolism pathways may affect inflammatory fluctuations in RA. This study identified the most robust core differential metabolites using the following criteria: 1) The criteria for selecting differential metabolites within each group were: P < 0.05 and Fold Change (FC) ≥ 1.5 or FC ≤ 1/1.5. Subsequently, the top 10 metabolites with the smallest P-values within each group were listed. Additionally, screening was performed using the VIP values from the OPLS-DA model, where a higher VIP value indicates a greater contribution of the metabolite to the group. 2) Clear RA-related biological functions (based on the above differential metabolite screening criteria, KEGG annotation, and existing literature). The final screening identified core differential metabolites such as Stizolamine and Cyanidin 7-arabinoside. However, as this study did not include independent cohort validation, the clinical value of these metabolites as biomarkers requires further confirmation. Currently, they can only serve as potential season-specific monitoring indicators. Future studies will clarify their diagnostic efficacy and prognostic value through expanded sample sizes, multicenter validation, and longitudinal follow-up. Based on the seasonally variable metabolites and inflammatory markers identified in this study, preliminary RA season-specific management recommendations can be proposed: 1) Disease monitoring: Increase RA disease monitoring frequency in spring (peak disease activity), with a focus on pro-inflammatory metabolite levels; maintain routine monitoring in autumn (lowest disease activity). 2) Treatment Adjustment: Winter may warrant supplemental DHA derivatives, while summer may require optimizing maintenance therapy in response to rising anti-inflammatory metabolites; 3) Relapse Prediction: Significant elevations in pro-inflammatory metabolites in patient serum may indicate potential inflammation relapse risk, necessitating timely intervention. However, these recommendations require further optimization through clinical validation with larger sample sizes to ensure feasibility and efficacy. In summary, this study confirms seasonal variations in metabolic characteristics among RA patients, which may be associated with dynamic regulation of lipid metabolism networks. These findings provide a partial metabolic perspective on understanding seasonal influences on RA, potentially offering directions for exploring season-specific precision medicine approaches (e.g., personalized drug targeting lipid metabolism, screening for season-related biomarkers). They also lay a foundation for developing novel anti-rheumatic treatment strategies. However, this study lacks data from healthy control groups. Future research should incorporate healthy controls and employ case-control designs to clarify RA-specific seasonal metabolic characteristics.

## 6 Limitations

However, we must also acknowledge certain limitations of this study. First, this study did not include healthy individuals or those with non-RA inflammatory diseases as controls. Consequently, it remains unclear whether the observed seasonal variations in lipid metabolism are specific to RA, and it is difficult to distinguish whether these patterns represent disease-specific pathological manifestations or are the result of general inflammatory or seasonal metabolic fluctuations. Second, although LC-MS/MS technology offers high sensitivity and resolution, it may still introduce some degree of error in metabolite identification and quantification. All patients in this study were from the Guiyang region (subtropical humid monsoon climate, with an annual temperature difference of approximately 19 °C and average annual humidity of about 77%). The unique climatic characteristics of this region (cold and humid winters, hot and humid summers), dietary patterns (high-fat, high-oil local cuisine), and lifestyle (reduced outdoor activity during winter) may influence seasonal fluctuations in the metabolome.” Significant variations in temperature, humidity, daylight duration, and dietary patterns exist across different regions (e.g., northern temperate monsoon climate, northwestern arid climate), suggesting potential heterogeneity in metabolic seasonality among RA patients. Future studies should conduct multicenter, cross-regional investigations involving RA populations from diverse climatic zones to validate the generalizability of these findings and identify region-specific metabolic regulatory factors. Additionally, this study focused solely on serum metabolites; metabolic changes in other tissues or bodily fluids may also significantly impact RA progression, necessitating future research to expand the scope of detection. Furthermore, individual responses to seasonal changes may vary, potentially influenced by factors such as genetic background, lifestyle, and comorbidities. Therefore, when applying the results of this study to clinical practice, it is essential to consider patients’ individual characteristics for more precise assessment and treatment. Further research could explore the specific associations between these season-related metabolic changes and clinical symptoms, as well as disease activity indicators. Additionally, long-term tracking of metabolic changes in the same group of patients across different seasons will help more accurately reveal the seasonal fluctuations in RA disease progression. In summary, the seasonal differences in RA serum metabolites identified using LC-MS/MS technology in this study represent a valuable starting point, but many questions remain to be addressed in future research to potentially bring new breakthroughs in the prevention and treatment of RA. 

## Data Availability

The original contributions presented in the study are included in the article/**Supplementary Material**. Further inquiries can be directed to the corresponding authors.
